# Tumor associated MUC1 carried by microvesicles is cross-processed by dendritic cells generating CD8+ T cell response

**DOI:** 10.1186/2051-1426-1-S1-P140

**Published:** 2013-11-07

**Authors:** Ilaria G Zizzari, Federico Battisti, Chiara Napoletano, Hassan Rahimi, Salvatore Caponnetto, Paola Martufi, Stefania Salvati, Morena Antonilli, Francesca Belleudi, Marianna Nuti, Aurelia Rughetti

**Affiliations:** 1 Department of Experimental Medicine, Sapienza University, Rome, Italy; 2 Department of Gynaecology and Obstetrics, Sapienza University, Rome, Italy; 3 Department of Molecular Medicine, Sapienza University, Rome, Italy

## 

The induction of an efficacious anti-tumor immune response (IR) requires the cross-processing and presentation of tumor antigen by Dendritic Cells (DCs). Block of endocytated tumor associated antigen (TAA) in the early compartments of the intracellular processing machinery shifts the IR towards a Th2 balance. MUC1 is one of the most relevant tumor associated glycoprotein expressed by epithelial cells and its immunogenicity is altered by the glycosylation profile. Moreover soluble MUC1 antigen has shown to be stucked in endolysosomal compartment of DCs thus inducing mostly a Th2 response. Objective of this study was to investigate whether glycosylation pattern and MUC1 bound to microvesicles could influence the antigen processing by DCs. MUC1 as soluble molecule, independently by the glycosylation profile, appears to be blocked in the pre-endosomal compartment. Receptor-mediated endocytosis pushes further the processing in the HLAII compartment. Cross-processing of MUC1 in HLAI compartment is observed only when MUC1 is carried by microvesicles (MUC1-MVs). Moreover only DCs stimulated with MUC1-MVs are able to induce IFN-γ production by MUC1 specific CD8+T cells. The distinct processing of the MUC1 membrane bound is accompanied by deglycosylation processes thus generating Tn-MUC1 immunogenic glycoforms. These results show that MUC1 undergoes to alternative processing pathways depending by its form of release (MVs vs soluble) thus modifying MUC1 immunogenicity. Moreover these experimental evidence can be important to design efficacious glycoantigen formulations for DC-based cancer vaccines.

